# Colonisation of *Oncidium* orchid roots by the endophyte *Piriformospora indica* restricts *Erwinia chrysanthemi* infection, stimulates accumulation of *NBS-LRR* resistance gene transcripts and represses their targeting micro-RNAs in leaves

**DOI:** 10.1186/s12870-019-2105-3

**Published:** 2019-12-30

**Authors:** Wei Ye, Jinlan Jiang, Yuling Lin, Kai-Wun Yeh, Zhongxiong Lai, Xuming Xu, Ralf Oelmüller

**Affiliations:** 1 Sanming Academy of Agricultural Sciences, Sanming, Fujian China; 2 Institute of Horticultural Biotechnology, Fujian Agriculture and Forestry University, Fuzhou, Fujian China; 3 Matthias-Schleiden-Institute, Plant Physiology, Friedrich Schiller University Jena, Jena, Germany

**Keywords:** *Oncidium*, *Piriformospora indica*, *Erwinia chrysanthemi*, microRNA, Resistance gene

## Abstract

**Background:**

*Erwinia chrysanthemi* (*Ec*) is a destructive pathogen which causes soft-rot diseases in diverse plant species including orchids. We investigated whether colonization of *Oncidium* roots by the endophytic fungus *Piriformospora indica* (*Pi*) restricts *Ec*-induced disease development in leaves, and whether this might be related to the regulation of nucleotide binding site-leucine rich repeat (NBS-LRR) *Resistance* (*R*) genes.

**Results:**

Root colonization of *Oncidium* stackings by *Pi* restricts progression of *Ec-*induced disease development in the leaves. Since *Pi* does not inhibit *Ec* growth on agar plates, we tested whether NBS-LRR *R* gene transcripts and the levels of their potential target miRNAs in *Oncidium* leaves might be regulated by *Pi*. Using bioinformatic tools, we first identified NBS-LRR *R* gene sequences from *Oncidium*, which are predicted to be targets of miRNAs. Among them, the expression of two *R* genes was repressed and the accumulation of several regulatory miRNA stimulated by *Ec* in the leaves of *Oncidium* plants. This correlated with the progression of disease development, jasmonic and salicylic acid accumulation, ethylene synthesis and H_2_O_2_ production after *Ec* infection of *Oncidium* leaves. Interestingly, root colonization by *Pi* restricted disease development in the leaves, and this was accompanied by higher expression levels of several defense-related *R* genes and lower expression level of their target miRNA.

**Conclusion:**

Based on these data we propose that *Pi* controls the levels of NBS-LRR *R* mRNAs and their target miRNAs in leaves. This regulatory circuit correlates with the protection of *Oncidium* plants against *Ec* infection, and molecular and biochemical investigations will demonstrate in the future whether, and if so, to what extent these two observations are related to each other.

## Background

Orchids such as *Oncidium*, *Phalaenopsis* and *Dendrobium* have high commercial value and are becoming globally important for the agro-industry. However, modern commercial orchid cultivars are selected by artificial pollination and multiplied by micro-propagation; as a consequence, the low gene diversity and large scale cultivation make them susceptible to pathogen infection causing great economic loss. For example, *Erwinia chrysanthemi* (*Ec*, also known as *Dickeya dadantii* or *Pectobacterium chrysanthemi*) is one of the soft rot pathogens [1, 2] which causes economic losses in a wide variety of crops and orchids, including *Oncidium* (cultivar *Onc.* ‘Gower Ramsey’, the most often commercialized cultivar in Taiwan, South East Asia and China) [3, 4]. Up to now, no resistance (*R*) genes against soft rot disease have been reported in orchids. Moreover, most orchids have long vegetative growth, and the long breeding cycle prevents an improvement of single characteristics via hybridization. Identification and characterization of *R* genes from the existing orchid germplasm resources would be helpful in breeding high-resistance orchid cultivars and in genetic engineering programs.

*Piriformospora indica* (*Pi*), originally isolated from the woody shrubs rhizosphere in an Indian desert, is a root-colonizing endophytic fungus with a broad range of host plants. It confers diverse beneficial effects on host plants by improving nutrition uptake [5, 6], promoting biomass production [7–9], stimulating the accumulation of secondary metabolites [10, 11], and strengthening resistance against biotic and abiotic stresses [12–16].

The nucleotide binding site-leucine rich repeat (NBS-LRR) resistance (R) proteins function as molecular switches. They are characterized by highly conserved motifs, including the P-Loop/Kinase-1a [GGV(I/M)GKTT], Kinase-2 [LVDDVW(D)], Kinase-3a (GSRIIITTRD) and GLPL [GL(F)PL(F)AL] motifs in all plant species [17]. R proteins recognize directly or indirectly pathogens’ effectors and trigger or suppress downstream defense responses in plants. Thus far, 149 and 480 genes for NBS-LRR R and LRR domain proteins were identified in Arabidopsis and rice, respectively [18, 19]. They are classified into two major groups: Toll/Interleukin-1 receptors (TIR) and non-TIR-NBS-LRR proteins, based on the N-terminal TIR or curly coiled-coil structure [20].

Micro-RNAs (miRNAs), small non-coding RNAs, silence gene expression at transcriptional and post-transcriptional levels, and participate in numerous processes including plant defense [21–23]. For example, *miR393* is a pathogen-associated molecular pattern-responsive miRNA which contributes to disease resistance, and pathogen effectors can suppress *miR393* accumulation to facilitate disease development [24, 25]. Additionally, miRNA-mediated *R* gene silencing plays an important role in the development of plant-microbial symbiosis and systemic resistance [26–28]. In *Medicago truncatula*, the expression levels of miRNAs such as miR399k^*^, miR1507, miR1510a*, *miR2678* and *miR5213*, which have been verified to regulate NBS-LRR *R* genes, were suppressed during the mycorrhiza formation; moreover, *miR5213* is only found in mycorrhizal plants [29].

In a previous study, we reported that colonization of *Oncidium* roots by *Pi* regulates a group of miRNAs and related target *R* genes [30]. 1083 miRNAs belonging to 56 families were detected in a transcriptomic library from *Pi*-colonized roots, but not from uncolonized roots. Furthermore, the expression patterns of miRNAs and their target genes during the symbiotic process showed significant changes during root colonisation. Especially, the miRNAs involved in auxin signaling functions and root development responded to *Pi* colonization. The work suggested that *Pi* promoted plant growth through regulating the expression level of miRNAs and their target genes. In the present work, we focus on the mechanism of pathogen resistance induced by *Pi*-colonization. The expression pattern of miRNAs and their target transcripts for NBS-LRR R proteins was investigated in *Ec*-infected leaves of *Pi*-colonized and uncolonized *Oncidium*. Our work revealed that root colonization by *Pi* activates the expression of NBS-LRR *R* genes in the leaves which correlated with an increase resistance against *Ec* infection. Furthermore, when a leaf is infected by *Ec*, *Pi* can suppress the accumulation of *Ec*-induced miRNAs in the leaves which results in high expression of their target *R* genes. The counteracting mechanism between *Pi* and *Ec* is discussed in the context of root-to-shoot signaling.

## Results

### Mining for NBS-LRR-type R genes in Oncidium

NBS-LRR R proteins play an important role in plant resistance against abiotic and biotic stress and act as a molecular switch to regulate defense in plant-microorganism interactions [31, 32]. In a previous study, we identified miRNAs which were significantly up-regulated in *Oncidium* roots in response to *Pi* colonization [30]. Closer inspection of these miRNAs uncovered that many of them are predicted to target *R* gene transcripts. This prompted us to investigate the regulation of *R* genes/transcripts and their potential target miRNAs in the tripartite interaction between *Oncidium*, *Ec* and *Pi* in greater details.

To identify *R* genes in *Oncidium*, we used the previously described transcriptomic datasets (accession: PRJNA428913, [30]). 24,616 deduced amino acid sequences from the transcripts were scanned for pfam NB-ARC HMM profiles (pfam: PF00931) by an E-value cut-off of < 1^− 40^ with the HMMER software. We identified 99 candidate *R* gene sequences using an E-value cut-off of < 1^− 2^. The proteins deduced from these sequences contained between 62 and 610 amino acids (average length: 229 amino acids) (Additional file 3: Table S1). Similar methods were used for the identification of *R* genes in *Phalaenopsis equestris* [33] and *Dendrobium officinale* [34] and resulted in 63 and 94 candidate sequences with the respective whole genomic sequence information.

Subsequently, the candidate R protein sequences from *Oncidium* were used for BLAST searches against the NCBI nr database using BLASTP. Ninety-six of the 99 candidates matched to known disease R proteins with 50.2 to 88.0% similarity (E-values: between 0 and 1.56^− 11^). Among them, 57 and 36 proteins were highly similar to R proteins from *D. officinale* and *P. equestri*, respectively (Additional file 1: Figure S1)*.*

NBS-LRR R proteins are characterized by their amino-terminal TIR domains or CC motifs, and a carboxyl-terminal LRR domain. The 99 candidate sequences were further analyzed with the InterProScan program on the BLAST2GO software. We identified 87 sequences with NB-ARC domains, 16 with CC motifs, 36 with LRR domains, and none with a TIR domain (Additional file 3: Table S1). TIR domains were also not detected in the predicted R protein sequences of *D. officinale* and *P. equestri*.

The NB-ARC motif-containing R proteins identified by the MEME analyses were identical to the *Oncidium* R proteins found with the InterProScan program. Eight types of major motifs, including the P-loop, RNBS-A-non-TIR motif, kinase-2, RNBS-B, GLPL, RNBS-C, RNBS-D and an MHD-like motif, were identified in *Oncidium* R proteins (Table 1). Among them, the P-loop, RNBS-A, RNBS-B and RNBS-C motifs showed the highest conservation. However, the GLPL was replaced by the GC/SPLAA motif in *Oncidium*. The same replacement was also found in *P. equestri,* but not in *D. officinale* where the original sequence GLPLAL/I was conserved. Furthermore, the MHDL motif was replaced by a MHD-like motif in *Oncidium*, which is also found in apple [35] and *Phalaenopsis* [33]. Finally, a highly conserved FxKxDLVRMW motif, located ~ 40 amino acids N-terminal to the MHD-like motif sequence, is also presented in *P. equestri* and *D. officinale*, but not found in other species including *Arabidopsis* [17], *Japonica* rice [18], *Populus trichocarpa* [36], soybean [37], *Solanum tuberosum* [38] and *Lotus japonicas* [39].

**Table 1:** The motifs of R protein sequences identified by MEME

No.	Best match	NBS motif	E-value
1	FCxxFxQDHxFDKDDLVRMW		9.1^− 310^
2	LsVVGH/MGGMKxTLLQHVY	P-loop	1.0^− 295^
3	MVxKLxGC/SPLAAKVIGGILN	GLPL	7.8^− 266^
4	SYxxLPxxLxxCFxFCxxFP	RNBS-D	8.3^−250^
5	FxVK/QxW/FV/ACVSxNFxAxxVIX	RNBS-A-non-TIR	1.4^−266^
6	xYKMHDLLHELAQS/EVSxxEx	MHD-like	3.1^− 256^
7	VLAPLxxGSS/LGSKxLITTRx	RNBS-B	2.2^−259^
8	DxGRcYFN/DILVxxSFFDEFx		9.9^− 245^
9	D/ExCLxLF/LxxH/YAFA/FGVENPDD	RNBS-C	2.2^− 243^
10	KRFLL/IVxDDI/VWExDExxWxN	Kinase-2	5.2^−234^

*If the bit value of the amino acid at this position is < 1, it is replaced by an x; conserved amino acid sequences are shown in bold letters

### Phylogenetic analysis of NB-ARC domain-containing R proteins from *Oncidium*

**Fig. 1:**
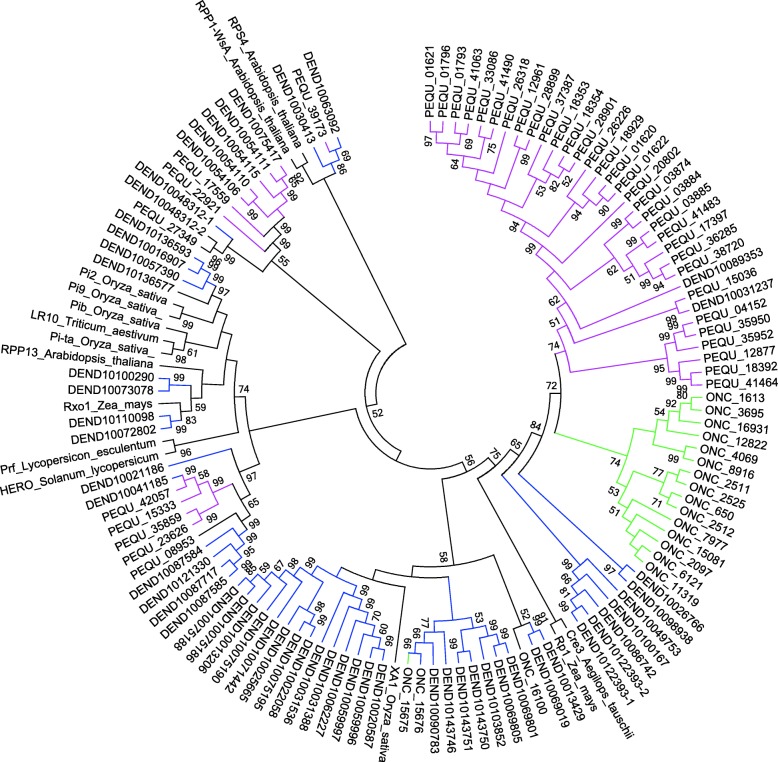
Phylogenetic analysis of R proteins of *Oncidium*, *Phalaenopsis equestris* and *Dendrobium officinale* orchids. Bootstrap Neighbor-Joint tree was constructed for the R proteins from *Oncidium* (ONC, green)*, Phalaenopsis equestris* (PEQU, red) and *Dendrobium officinale* (DEND, blue) using MEGA6.01 and the respective NBARC domains (from P-loop to MHD-like domain) (Fig. 2). The sequences were compared to 15 known R protein sequences: TNL: RPP-1 (AAC72977), RPS4 (BAB11393); NL: Pi9 (ABB88855)*,* Pi2 (ABC94599)*,* Pib (BAA76281); XNL: Prf (U65391); CNL: Rp1 (AAP81262), RXO1 (AY935244), Xa1 (BAA25068), Pita (AAK00132), Cre3 (AAC05834), Lr10 (aaq01784), RPM1 (NP187360), RPP13 (AF209732) and HERO (CAD29728)

To study the evolutionary relationships of *Oncidium* R proteins, a Neighbor-Joining phylogenetic tree was built based on the conserved NB-ARC domain (from P-loop to MHD-like motif) by using the MEGA6.06 software. Eighteen of the 99 *Oncidium* R protein sequences contain the complete NB-ARC domain. Together with 15 well-known R protein sequences from other species, they were used for the phylogenetic analysis (Fig. 1). As expected, two well-known TIR type R proteins were grouped into the TNL clade, while the *Oncidium* sequence*s* were grouped into the non-TNL clade together with the well characterized R proteins from the other species. Furthermore, 15 of the 18 *Oncidium* R proteins form an independent clade and show only a distant relationship to the known XA1 (rice), Cre3 (wheat) and Rp1 (maize) R proteins. The result suggests that the diversification of *R* genes in *Oncidium* and other orchids has a unique evolutionary history.

### Prediction of miRNA target sequences in *Oncidium R* gene mRNAs

The putative 99 *R* gene sequences were used to identify target sequences for miRNAs which had been identified previously in *Pi*-colonized *Oncidium* (Accession: SRP031471, as described in [30]), by using the psRNAtarget search program with E value < 3. We performed a BLAST search against the miRBase1.9 (plant section) which contained 4562 miRNA sequences. As shown in the Table 2 and Additional file 5: Table S3, 43 of the 99 *R* gene sequences were positively predicted to be targeted by 46 miRNAs. Among them, *miR1507*, miR1510a*, *miR2118* and *miR482/472* were commonly reported in controlling NBS-LRR *R* gene expression in *M. truncatula* [29], cotton [40] and potato [41, 42]. Furthermore, several of the identified miRNAs are predicted to target multiple *R* messages, such as *miR1514* (16 *R* mRNAs), miR1510a* (14 *R* mRNAs), *miR5246* (13 *R* mRNAs) and *miR5654* (12 *R* mRNAs). Therefore, it appears that the mRNA levels of many *R* genes might be regulated by more than one miRNA species.

**Table 2:** Predicted *R* genes and the targeting miRNAs

Target	Counts (RPKM)		Annotation	Predicted regulator miRNA
	CK	P		
*Onc 235*	7.9	15.4	uncharacterized protein LOC100279616	*miR2088*, *miR5654*
*Onc 649*	12,.0	14.0	OsJNBa0083D01.14	*miR1514*
*Onc 650*	7.8	3.0	hypothetical protein OsI_07084	miR1510a*, *miR1514*
*Onc 651*	0	14.7	putative disease resistance protein I2	*miR1514*
*Onc 1207*	59.9	93.6	hypothetical protein OsI_15587	miR1510a*
*Onc 1471*	60.9	81.6	putative disease resistance protein RGA4	*miR1514*, *miR5654*
*Onc 1537*	86.2	85.7	hypothetical protein OsJ_14506	*miR1514*, *miR2088*, *miR5654*
*Onc 1613*	13.2	27.5	disease resistance protein I2	*miR1510*, *miR529g*
*Onc 1615*	28.1	15.7	NBS-containing resistance-like protein	*miR5246*
*Onc 1618*	0.0	10.7	hypothetical protein OsI_07084	miR1510a*
*Onc 1724*	89.8	99.4	hypothetical protein SORBIDRAFT_08g020630	*miR1514*
*Onc 2107*	8.5	15.0	putative disease resistance protein	*miR156k*, *miR2088*, *miR5654*
*Onc 2524*	18.5	27.2	putative disease resistance protein RGA2	*miR2088*
*Onc 2555*	24.6	60.0	putative disease resistance protein RGA4	*miR1514*
*Onc 3695*	11.7	22.9	CC-NBS-LRR R protein	miR1510a*, *miR529g*
*Onc 4069*	36.1	32.0	hypothetical protein OsI_15587	miR1510a*, *miR1514*, *miR2088*, *miR5246*, *miR5654*
*Onc 4126*	0.0	17.4	CC-NBS-LRR R protein	miR1510a*
*Onc 4434*	16.5	10.8	hypothetical protein VITISV_025836	*miR2088*, *miR5654*
*Onc 4722*	8.6	20.2	hypothetical protein OsI_07084	miR1510a*, *miR894*
*Onc 4724*	41.8	107.4	NB-ARC domain-containing protein	miR1510a*, *miR857*
*Onc 5026*	0.0	15.7	disease resistance protein I2	*miR166c*
*Onc 5046*	26.3	70.0	uncharacterized protein LOC100279616	*miR5654*
*Onc 5277*	25.9	52.6	hypothetical protein OsI_07084	*miR1510, miR5246*
*Onc 5425*	41.1	48.2	putative disease resistance protein	*miR1514, miR5654*
*Onc 5583*	119.0	203.5	hypothetical protein VITISV_018147	*miR482*
*Onc 6091*	5.2	17.7	putative disease resistance protein	*miR156k*, *miR2088*, *miR5654*
*Onc 6121*	3.2	7.5	hypothetical protein OsI_15587	*miR1514*, *miR5246*
*Onc 7005*	58.9	62.4	putative disease resistance RPP13 protein	*miR1514*
*Onc 7221*	20.1	50.3	hypothetical protein OsI_07084	miR1510a*
*Onc 7977*	32.2	25.1	putative disease resistance protein	miR1510a*, *miR5246*
*Onc 8764*	0.0	19.9	OsJNBa0083D01.14	*miR5654*
*Onc 8916*	23.4	40.2	hypothetical protein OsI_07084	*miR156k*, *miR5246*
*Onc 9873*	0.0	15.9	NB-ARC domain containing protein	miR1510a*, *miR5246*
*Onc 11,319*	9.1	10.6	putative disease resistance protein RGA3-like	*miR1514*, *miR5246*
*Onc 12,822*	31.6	53.9	hypothetical protein OsI_15587	miR1510a*, *miR156k*
*Onc 13,214*	0.0	11.9	NBS-LRR protein	*miR1514*, *miR529g*
*Onc 15,037*	0.0	9.0	NB-ARC domain containing protein	miR1510a*
*Onc 15,081*	26.7	20.8	putative disease resistance RPP13 protein	*miR5246*
*Onc 16,931*	7.8	21.2	hypothetical protein OsI_15587	miR1510a*, *miR1514*, *miR166c*, *miR2088*, *miR5246*, *miR529g*
*Onc 19,773*	6.3	0.0	putative disease resistance protein At3g14460	*miR2088*, *miR5654*
*Onc 19,900*	0.0	3.4	OSIGBa0148A10.13	*miR5654*
*Onc 20,607*	0.0	6.5	hypothetical protein OsJ_14506	*miR838*
*Onc 28,117*	9.1	3.6	NBS-LRR R protein	*miR2118*

Count normalized by RPKM (reads per kilobase million). CK, control RNA from uncolonized *Oncidium* roots; P, RNA from *Pi*-colonized *Oncidium* roots

### *Pi* protects *Oncidium* against *Ec* infection, but does not inhibit *Ec* growth on agar plates

Further on, we wanted to assess how the expression of the identified *R* genes is related to *Oncidium* defence against *Ec*, and also if it could be modified by colonization with *Pi*. First of all, orchid cuttings were inoculated with *Pi* for 2 weeks. Microscopic observation demonstrates that *Pi* mycelia and spores were present in the cortex and velamen of *Oncidium* roots (Additional file 2: Figure S2A and B), indicating successful colonization. Subsequently, the second leaf of *Pi-*colonized and uncolonized control cuttings was infected with *Ec* bacteria, as described previously [3]. The treated plants were monitored on the 1st, 3rd, 7th and 21th day after infection (dai) by visible examination and counting the cell number of the pathogenic bacteria. At 1st dai, *Ec* caused obvious necrosis on the inoculated leaves of both *Pi*-pretreated and control cuttings. Until the 3rd dai, control plants not pre-treated with *Pi* showed pathogen-induced disease symptoms in the infected and the neighboring uninfected leaf, as well as in the connecting stems. However, the disease symptoms in the *Pi*-colonized cuttings were restricted to the infected leaf. Until the 7th dai, the control without *Pi* displayed necrosis in the whole plant, i.e. in all leaves, stems and roots. Moreover, *Ec* grew widespread on the MS medium and started to infect the neighboring healthy plants via the roots. In contrast, bacterial growth in *Pi*-colonized plants was mainly detectable on the inoculated leaf, and little infection was visible in neighboring leaves. No *Ec* bacteria could be observed on the MS medium. Even at the 21th dai, the *Pi*–colonized plants continued to grow (Additional file 2: Figure S2D), while the control plants were dead. These results indicate that *Pi* confers resistance against *Ec* infection and inhibits growth and propagation of the bacterium.

Better performance of the *Pi*-colonized plants after *Ec* infection might be caused by a direct inhibition of *Ec* growth, or by stimulating plant immunity. To investigate the *Pi*-mediated mechanism, both microbes were co-cultured on an agar plate*.* As shown in Additional file 1: Figure S1E, there is no inhibition zone between *Pi* mycelium and the *Ec* colony. On the contrary, *Ec* actively inhibits the mycelial growth of *Pi* hyphae, as visible by comparison of the growth of *Pi* on plates without the bacterium (Additional file 2: Figure S2E). This suggests that better performance of *Pi*-colonized *Oncidium* plants after *Ec* infection is probably not caused by direct inhibition of the bacterial growth in the plant. Rather, the acquired resistance might be caused by *Pi*-stimulated defense in the host. This differs from a previously report in which *Pi* protected *Arabidopsis* seedlings from *Verticillium dahliae* infection by inhibiting *V. dahliae* growth both on plates and in the plant [14].

**Fig. 2:**
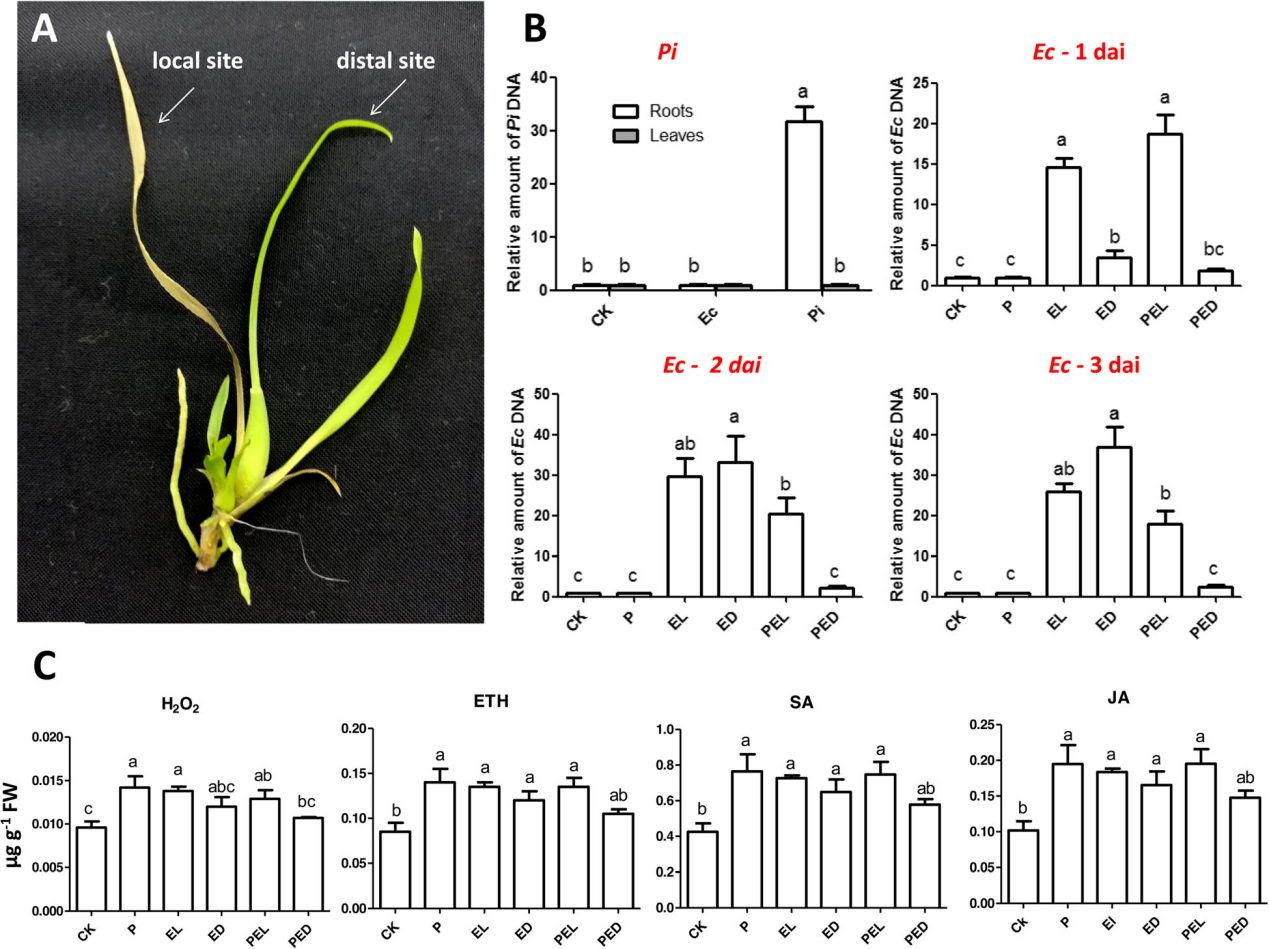
Detection of the pathogen in leaf tissues in *Pi*-colonized/−uncolonized *Oncidium*. **a** *E. chrysenthemi* (*Ec*) was locally inoculated on the second leaf of *Pi*-colonized/−uncolonized cuttings, respectively. Local and distal leaves were collected separately. **b** *Ec* DNA levels in leaves were detected by qPCR of 16S rDNA 1, 2 and 3 days after infection, *Pi* DNA in leaves and roots were detected with EF-hand DNA primer pair 10 days after inoculation, data represent the means ± SE of 3 replicates and were normalized to the plant *ACTIN* DNA level, values with the same letter were not significantly different (*p* < 0.05). **c** Levels of endogenous salicylic acid, jasmonic acid, ethylene and H_2_O_2_ 24 h after infection of the leaf with *Ec*. Data represent the means ± SE of 3 replicates, values with the same letter were not significantly different (*p* < 0.05). *PI*: qPCR for *Pi* and *Ec* DNA in roots/leaves of *Pi*-colonized cuttings. CK: uncolonized plants. *EC*1d, *EC*2d and *EC*3d indicates the detection of the presence *Pi* and *Ec* in *Pi*-colonized/−uncolonized plants 1, 2, or 3 days after *Ec* infection, relative values normalized to the plant *ACTIN* DNA level. CK: control plant. P: *Pi*-colonized plants; (P)EL: local infected leaf of *Pi*-uncolonized (EL) or -colonized (PEL) plants inoculated with *Ec*. (P)ED: distal leaves of *Pi*-uncolonized (ED) or –colonized (PED) plants inoculated with *Ec*

To investigate how *Pi* protects *Oncidium* plants against *Ec*-induced disease symptom development, we determined growth and propagation of the pathogen in the plant by real-time quantitative PCR (qPCR). *Ec*-inoculated leaves and the non-infected neighboring leaves (Fig. 2a) were separately harvested. We used 16S rDNA to detect the propagation of *Ec* in the different leaves and compared the results of *Pi*-colonized and un-colonized *Oncidium* plants. As shown in Fig. 2b, in the absence of *Pi*, *Ec* can be detected in the local infected leaf (EL) and the distal leaf (ED) 2th dai, confirming that the pathogen is highly infectious to *Oncidium*. In contrast, *Ec* could only be detected in the local infected leaf of *Pi*-colonized plants (PEL, Fig. 2b), and was not detectable in the distal leaf of the same plant (PED, Fig. 2b). We also examined the salicylic acid (SA), jasmonic acid (JA) and ethylene (ETH) levels and compared the hormone levels with those for H_2_O_2_ 24 h after *Ec-*infection in *Pi*-colonized and uncolonized plants. The results of the ELISA assays indicate that the hormone and H_2_O_2_ levels increased 1.5–2.0-fold in the *Ec*-infected and none-infected leaves of *Pi*-colonized and uncolonized plants (Fig. 2c), but the effects were always lower in the distal leaf of *Pi*-colonized plants although not always significantly (PED, Fig. 2c). These results indicate that the microbes control hormone and H_2_O_2_ responses in the leaves. Apparently, in tissues where *Pi* restricts propagation of the pathogen and disease development, the phytohormone and H_2_O_2_ levels are lower (Fig. 2c, PED).

### Colonization of *Oncidium* roots by *Pi* affects the expression of *R* genes and the accumulation of their target miRNA levels in leaves

Analysis of previously performed expression profiles [30] demonstrated that most of the 43 *R* mRNA levels predicted to be targeted by miRNAs responded to *Pi* colonization in *Oncidium* roots (Table 2 and Additional file 4: Table S2)*.* Transcripts for 24 *R* genes were up-regulated and for 8 *R* genes down-regulated by the fungus. Transcripts for 10 *R* genes could only be detected in *Pi*-colonized plants. One *R* gene was only expressed in uncolonized roots. However, it is worth noting that almost all miRNA levels which are predicted to target the messages of *R* genes were present in low abundance in our high-throughput sequencing data (Additional file 4: Table S2).

**Fig. 3:**
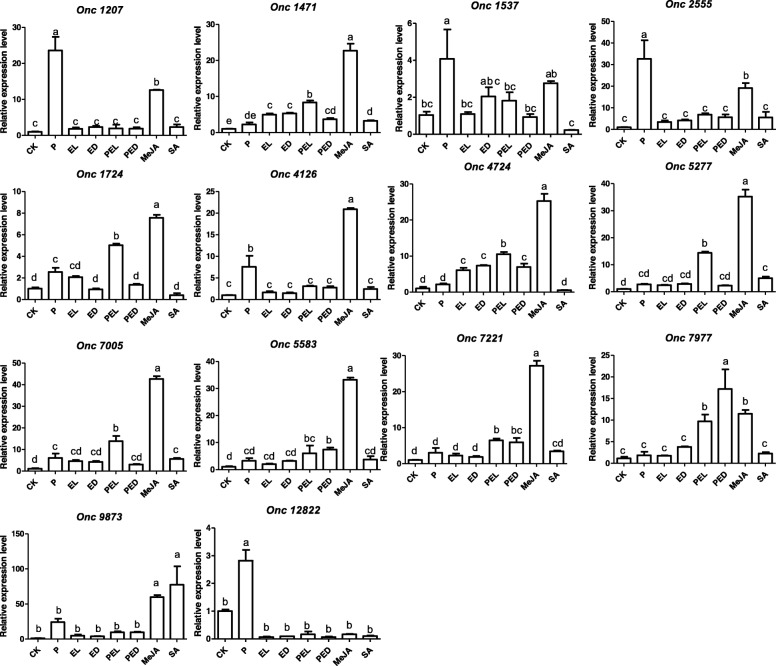
Expression of *R* genes after *Ec* infection of leaves of *Pi*-colonized or -uncolonized *Oncidium*. Expression levels of *R* genes 24 h after *Ec* infection of *Pi*-colonized (2 weeks) or –uncolonized *Oncidium* plants. 24 h after infection, the leaves were harvested for qRT-PCR analyses. CK: control plant without *Pi* colonization and *Ec* infection. P: *Pi*-colonized plants; (P)EL: local infected leaf of *Pi*-uncolonized (EL) or -colonized (PEL) plants. (P)ED: distal leaves of *Pi*-uncolonized (ED) or –colonized (PED) plants. SA: leaves treated with 1.0 mM salicylic acid for 24 h. MeJA: leaves treated with 0.1 mM methyl jasmonic acid for 24 h. Data represent the means ± SE of 3 replicates and were normalized to the *Oncidium ACTIN* mRNA level, values with the same letter were not significantly different (*p* < 0.05)

As shown in the Additional file 1: Figure S1 and Additional file 2: Figure S2, the *Pi*-colonized *Oncidium* showed increased resistance against *Ec* infection in the leaf tissues. This suggests that defense information is transmitted from roots to leaves. To investigate the expression levels of *R* genes and the accumulation of miRNAs in response to either *Pi* colonization or *Ec* infection or both, qPCR was performed with RNA preparations from leaves for all 43 *R* genes predicted to be targeted by miRNAs (Table 2 and Additional file 4: Table S2). As shown in Fig. 3, 7 *R* genes (*Onc1207, Onc1537, Onc1724, Onc2555, Onc4126, Onc12822* and *Onc7005*) were significantly up-regulated in the leaves of *Oncidium* plants which were colonized by *Pi* (Fig. 3, P). This is consistent with our previous results from the high-throughput sequence data and suggests that signals transmitted from the *Pi-*colonized roots up-regulate these *R* genes in the leaves, although they were not yet exposed to any threat.

Twenty-four h after leaf infection by *Ec*, *Onc1471* and *Onc4724* were induced in the infected leaf (Fig. 3, EL, PEL) and the neighboring uninfected leaf (Fig. 3, ED and PED) of both colonized and uncolonized plants (Fig. 3, CK and P). However, the transcript levels for the *R* genes *Onc1471*, *Onc1724*, *Onc4724*, *Onc5277*, *Onc7005*, *Onc7221* and *Onc 79*77 were higher in *Ec*-infected leaves of *Pi*-colonized plants (Fig. 3, PEL) than in the leaves of *Pi*-uncolonized plants (Fig. 3, EL). Furthermore, the expression of the *R* genes *Onc5583, Onc7221* and *Onc7977* in none-infected neighboring leaves were higher in *Pi*-colonized *Oncidium* than in the *Pi*-uncolonized control plants (Fig. 3, PED and ED). These data suggest that the *R* transcript levels in the leaves respond to *Pi* colonization of the roots.

### Defense hormones in the resistance response

Stein et al. [43] demonstrated that JA signalling and the cytoplasmic, but not nuclear localization of NPR1 is required for *Pi*-induced resistance against powdery mildrew infection. In order to test whether defence hormones are involved in the *Oncidium* resistance response against *Ec*, leaves were treated with 1 mM SA or 0.1 mM MeJA. Interestingly, all *R* genes (except *Onc 12,822*) are significantly up-regulated after treatment with 0.1 mM MeJA (Fig. 3, MeJA), but only 4 *R* genes (*Onc1471, Onc5277, Onc7055* and *Onc9873*) were up-regulated after treatment with 1 mM SA (Fig. 3, SA)**.**

**Fig. 4:**
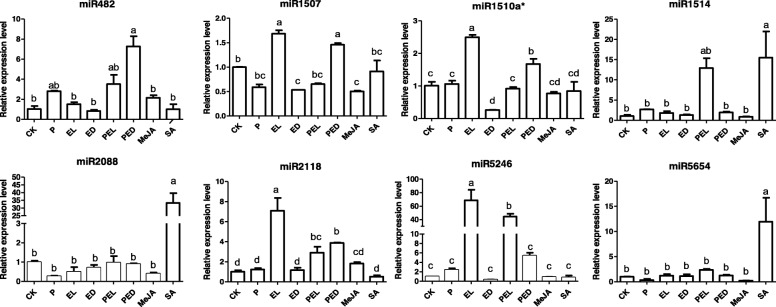
Expression of miRNAs after leaf infection of *Pi*-colonized or –uncolonized *Oncidium* with *Ec*. miRNA levels in *Pi*-colonized (2 weeks) or –uncolonized *Oncidium*. 24 h after *Ec* infection, the leaves were harvested for qRT-PCR analyses. CK: control plant without *Pi* colonization and *Ec* infection. P: *Pi*-colonized plants; (P)EL: local infected leaf of *Pi*-uncolonized (EL) or -colonized (PEL) plants inoculated with *Ec* for 24 h. (P)ED: distal leaves of *Pi*-uncolonized (ED) or –colonized (PED) plants inoculated with *Ec* for 24 h. SA: leaves treated with 1.0 mM salicylic acid for 24 h. MeJA: leaves treated with 0.1 mM methyl jasmonic acid for 24 h. Data represent the means ± SE of 3 replicates and were normalized to the *U6* snRNA level, values with the same letter were not significantly different (*p* < 0.05)

Next, we examined the levels of the identified miRNAs in response to *Pi*-colonization in *Oncidium* leaves. In contrast to the responses of the *R* mRNA levels, 7 miRNA levels did not change in the leaves when the roots were colonized by *Pi*. The only exception is the *miR482* level which was always higher in the leaves of *Pi*-colonised plants (Fig. 4, P). *Ec* stimulated the *miR1507*, miR1510a^*^, *miR2118* and *miR5246* levels in the infected leaves, and the effect was no longer detectable in the neighboring, none-infected leaves (Fig. 4, EL and ED). Up-regulation of these miRNA levels in the *Ec-*infected leaves was reduced when the roots were colonized by *Pi* (Fig. 4, EL and PED). Thus, the beneficial fungus influences the *Ec*-induced miRNA levels in the leaves.

Moreover, quite different from the *R* mRNAs, none of the miRNAs responded to exogenous application of MeJA (Fig. 4, MeJA), similar to the results obtained by *Pi* colonization. However, the *miR1514*, *miR2088* and *miR5654* levels were significantly up-regulated after SA treatment (Fig. 4, SA).

## Discussion

### *Pi* protects *Oncidium* against *Ec* infection

**Fig. 5:**
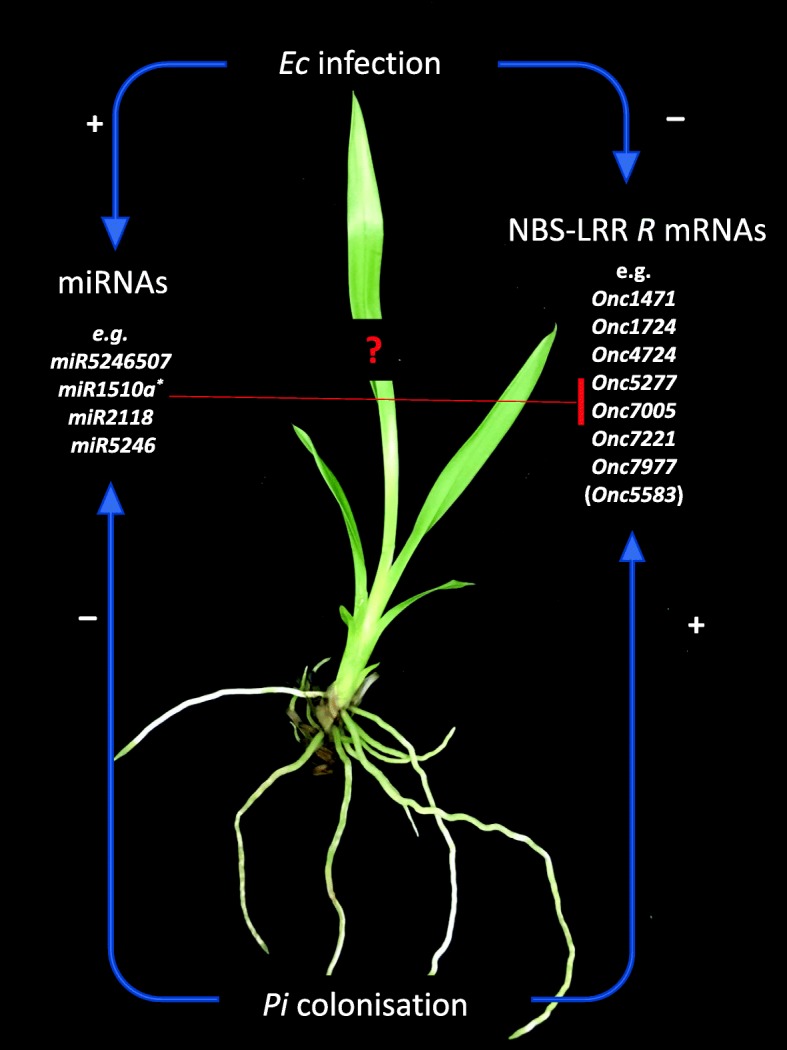
A model describing the regulation of miRNA and NB-LRR *R* mRNA levels in *Oncidium* leaves after *Ec* infection and root colonization by *Pi*

As reported previously for several pathosystems [44, 45], colonization of the roots by *Pi* confers resistance against leaf pathogens [13, 15, 16, 46–48]. We demonstrate that the severe disease symptom development induced by *Ec* in *Oncidium* leaves is partially restricted when the roots are colonized by *Pi*. *Ec* infection stimulates the accumulation of the defense hormones SA, JA and ETH in the leaves (Fig. 2c). The elevated hormone levels presumably participate in activating *R* gene expression which might participate in restricting disease development and propagation of the pathogen. Our data suggest that the regulation of NBS-LRR *R* genes and their related miRNA levels in the leaves could play a role in *Pi*-induced resistance against *Ec* infection, however, we did not provide any direct evidence for a link between the regulation of the *R* mRNA / miRNA levels and the disease symptom development in this study. Since *Ec* produce quite different virulence factors, proteins and metabolites (cf. below) which cause the disease symptoms in the infected plants, it is likely that additional plant defense compounds, mechanisms and strategies are involved in the host response. The *R* genes and miRNAs investigate here were identified in transcriptomic datasets generated from *Pi*-colonized *Oncidium* roots [30]. If they participate in the disease resistance phenotype, a possible and testable scenario could be that *Ec* counteracts the plant defense response by promoting the accumulation of miRNAs against the *R* messages (Fig. 5). Signals from the *Pi*-colonized roots might restrict miRNA accumulation in the leaves and thus support the plant defense against *Ec* infection (Figs. 3 and 5). The beneficial effect of *Pi* can be clearly seen by the restriction of *Ec* propagation in the non-infected neighboring *Oncidium* leaves. However, since the levels of all three defense-related phytohormones increased in response to *Ec* infection, we did not observe specific phytohormone effects, which allow allocation of the plant response to the JA/ETH-based defense against necrotrophs or the SA-based defense against biotrophs. This might be due to the massive destruction of the *Ec*-infected tissue which is associated with cell death processes and a collapse of a coordinated activation of the host defense system against the propagating pathogen. *Ec* is an opportunistic necrotrophic pathogen that does not appear to invade host cells internally in the pathogenic phase [49]. The bacteria remain in the intercellular spaces of infected plant tissue and use several secretion systems to inject virulence factors into host cells. Well-studied virulence determinants are also extracellular enzymes such as pectate lyase, pectinase, and cellulase; siderophore-dependent iron uptake systems, as well as the *sap* and *msrA* genes [49–55]. In addition to causing local disease, the bacteria enter vascular elements of infected plants, thereby moving rapidly through the host [50, 56–59]. This is consistent with the observed rapid collapse of the host defense system and highlights the importance for the search for strategies to restrict *Ec* propagation in infected plants. We propose that signals derived from *Pi-*colonized roots might be an interesting tool to control *Ec*-induced soft rod, wilts and blight diseases [49].

### *Pi* suppressed the accumulation of *Ec*-induced miRNAs

The involvement of miRNA in the regulation of host immune responses following fungal exposure has been described in many systems [60]. Interestingly, in human and animals, pathogen-induced changes in expression profiles have identified the same critical miRNAs which are also involved in inflammation and allergy responses [60], suggesting a broad conservation in the mechanisms. In plants, miRNAs play important roles in immune responses and defense gene activation [61–66]. Together with siRNAs (small interfering RNAs), they guide sequence-specific silencing of genes, and recognize repetitive DNA and virus nucleic acids through base complementarily [67]. In our study, we discovered miRNAs from transcriptomic datasets, which were predicted to silence *R* genes encoding NBS-LRR R proteins (Fig. 4). We showed that the *miR1507*, miR1510a*, *miR2118* and *miR5246* levels were induced after *Ec* infection (Fig. 4, EL), and this stimulation was either completely or partially prevented when the roots were colonized by *Pi* (Fig. 4, PEL). More importantly, the transcript levels for the *R* genes *Onc1471*, *Onc1724*, *Onc4724*, *Onc5277*, *Onc7005*, *Onc7221* and *Onc 79*77 were higher in *Ec*-infected local leaves of *Pi*-colonized plants (Fig. 3, PEL) than in the leaves of plants without *Pi* colonization (Fig. 3, EL). This suggests that *Pi* in and around the roots repress miRNAs accumulation in the leaves to maintain relative high *R* genes levels. Notably, similar results also have been reported for fusiform rust gall development in *Pinus taeda*, which is controlled by the inhibition of miRNA biosynthesis for target *R* mRNAs [68]. Besides, during early stage of nodulation, miRNAs as *miR482*, *miR1507*, *miR2118* accumulate to avoid plant immunity responses against the colonizing microbes [26, 29, 69]. Although there is little known about miRNAs and *R* genes involved in resistance effects conferred by *Pi*, our data demonstrate that they might be important regulatory components for disease control. The control of *R* mRNAs by the miRNAs is based on bioinformatic predictions of target sequences and similarities described for other pathosystems and requires experimental verifications. However, for some miRNAs, the molecular mechanisms have been investigated. During symbiosis development, miR1510a*-mediated cleavage was confirmed by degradome analyses and *miR1507*, *miR5213* and *miR2118* are predicted to target *R* genes [29]. The participation of *miR482*, *miR1705* and *miR2118* in the regulation of NB-LRR *R* transcripts was confirmed by 5′-RACE [41, 42, 70]. *miR1514*, which was reported to target *R* genes in *Oncidium* ([30] and ref. therein), was shown to target NAC-transcription factor *NAM* messages by degradome analyses [71]. *miR5654* targets transcripts of a *MYB* transcription factor [72], but this has not been confirmed experimentally. No experimental evidence for the mechanism has been shown yet for *miR5246* and *miR2088*. Furthermore, some miRNAs might also be involved in other responses, such as *miR482,* which is not stimulated by *Ec,* but by *Pi,* or *miR2088* and *miR5654*, which do not respond significantly to both microbes (Fig. 4). Furthermore, the *miR1507*, miR1510a*, *miR2118* and *miR5246* levels were repressed by *Pi* in the infected leaves, but not in the neighboring leaves. A possible explanation could be that the slower progression of the *Ec*-induced disease development in plants with *Pi*-colonized roots did not yet result in the activation of the R/miRNA-based defense mechanism, because the pathogen titer in the distal leaves of the *Pi*-pretreated plants is too low. Finally, since miRNAs target multiple *R* messages, such as *miR1514* (16 *R* mRNAs), miR1510a* (14 *R* mRNAs), *miR5246* (13 *R* mRNAs) and *miR5654* (12 *R* mRNAs), and a particular *R* mRNA can be targeted by different miRNA species, the results shown in the Figs. 3 and 4 provide only a basis for future investigations.

### Root-to-shoot information transfer

Better protection of the leaves against *Ec* infection by *Pi* requires root-to-shoot information transfer. Induced systemic resistance (ISR) is well established [73] and Stein et al. [43] demonstrated for *Pi* that JA signalling and the cytoplasmic, but not nuclear localization of NPR1 is required for resistance against powdery mildew *G. orontii* infection. The JA-insensitive mutants *jasmonate-resistant 1* (*jar1–1*) [74] and *jasmonate-insensitive 1* (*jin 1*) [75] as well as the null mutant *npr1–1* [nonexpressor of pathogenesis-related (PR) genes 1, also known as NIM1] [76] are compromised in *Pi*-mediated resistance [43]. The ISR is independent of SA and SA signalling [43, 73], since *NahG* plants expressing a bacterial salicylate-hydroxylase [77] and the *npr1–3* mutant, lacking the nuclear-localisation signal, were not affected in *Pi-*mediated resistance to *G. orontii* [73]. Whether the protective function of *Pi* against *Ec* infection in *Oncidium* leaves is mediated by a JA-dependent ISR, requires studies with plant hormone mutants, which are not available at present for *Oncidium*. Numerous other mechanisms are also possible. Symbiosis-specific compounds from *Pi* might travel from the roots to the leaves, the beneficial fungus might influence the metabolomic stage, or the transport efficiency of defense relevant compounds, to mention a few.

The increase in ETH emission after *Ec* infection demonstrates that this phytohormone is also involved in the defense response. ETH is involved in ISR conferred by *Pseudomonas fluorescens* WCS417r, [78, 79], and also Nie et al. [80] demonstrated that ISR against *Botrytis cinerea* by *Bacillus cereus* AR156 is mediated through a JA/ETH- and NPR1-dependent signaling pathway in Arabidopsis. However, ISR mediated by *P. fluorescens* CHA0r against *Peronospora parasitica* is independent of the ETH receptor ETR1 and the downstream signaling component EIN2 [81–83]. It appears that also *Pi*-mediated resistance does not require ETH signaling ([84], and ref. therein). Altered phytohomone levels in the leaves were proposed to suppress host immunity or to prime the aerial parts for better resistance against pathogen attack (cf. [43, 44, 46] and ref. therein, [85]). Stimulation of JA and JA-responsive genes by *Alternaria brassiacae* infection was strongly inhibited when the plants are colonized by *Pi* [86, 87], and the involvement of jasmonate signalling has been well confirmed for *Pi*-mediated ISR responses [43, 46, 88]. A similar ISR mechanism has been shown for a non-pathogenic *Rhizobium radiobacter* strain which forms a symbiotic interaction with *Pi* [89], and the authors proposed that the beneficial activity assigned to *Pi* may be at least partly allocated to its symbiotic bacterium. Systemic signals also stimulate defense-related responses in distal, not *Pi*-colonized root areas which inhibit secondary colonisation of the roots by the fungus [90]. Kinetical studies after infection of *Oncidium* with *Ec* in the presence or absence of *Pi* will shine light on the role of ETH in this system.

ISR is characterized by a weak or not detectable systemic regulation of defense-related transcripts in the absence of a challenging pathogen [91, 92] and only after a pathogen attack a stronger defense response was observed [cf. 43, 73]. A similar response was described for rhizobacteria-induced ISR in Arabidopsis [93]. Since *R* genes which respond to *Pi* also respond to MeJA application, it is tempting to speculate that the information flow from the roots to the shoots is based on a JA-dependent information flow. However, this requires more detailed kinetic analyses and a better understanding of the connection between *R* mRNA/miRNA and phytohormone levels (cf. Discussion in [94]).

Besides the involvement of phytohormones in *Pi*-induced systemic resistance responses, Felle et al. [95] showed that the beneficial fungus induces fast root-surface pH signaling which primes systemic alkalization of the leaf apoplast upon powdery mildew infection. Rapid propagation of information within the plant body has also been associated with combined electric, Ca^2+^ and ROS waves [96], and an Arabidopsis mutant which is unable to respond to *Pi* and fails to confer resistance to the pathogen in systemic tissue is impaired in all three responses [16], and ref. therein).

*Pi* has also been reported to directly inhibit growth of pathogens, such as of *Verticillium dahliae* on agar plates and in colonized Arabidopsis roots [14]. Since growth of *Ec* was not inhibited by *Pi* on agar plates, the protection of colonized *Oncidium* orchids against *Ec* propagation in the leaves is likely caused by a *Pi*-induced root response, rather than a direct inhibition of the propagation of the pathogenic bacterium.

*Ec* has a wide host range, and rapidly kills host tissues mainly by type II secreted macerating isoenzymes. Virulence effector proteins secreted by the type III secretion system may be less important for the disease development [97, 98]. During *Ec* infection in plants, accumulation of reactive oxygen species [99, 100] and phenolic compounds [101], as well as the expression of JA-, SA-, abscisic acid- and ETH-responsive defense genes have been observed [100]. In *Phaleanopsis* [4], besides *WRKY* and *MYB* genes, also NBS-LRR *R* genes responded to *Ec*. To date, no monogenic resistance mechanism has been described for orchids [100]. We propose that NBS-LRR *R* genes are targeted and enhanced to express by the signals from *Pi*-colonized roots to restrict *Ec* growth in the leaves. In parallel, repression of their target miRNAs occurs.

## Conclusion

These results indicated that *Pi* and MeJA promotes *R* gene expression in both local and distal leaves of *Oncidium*, while *Ec* and SA triggers the accumulation of their target miRNAs. It appears that *Ec* prevents *R* mRNA accumulation by stimulating the accumulation of their miRNAs in *Oncidium*, and *Pi* counteracts this effect (Fig. 5). How these regulatory processes are related to the protection of the plants against the bacterial infection, remains to be investigated.

## Methods

### Growth of the plant and microbes, co-cultivation and infection procedure

*Oncidium* (cultivar *Onc.* ‘Gower Ramsey’), a commercialized hybrid orchid, was originally obtained from the flower market in Fouzhou, China, the morphology and biology characteristics were identified [102]. The flower stalk buds were propagated at the Institute of Horticultural Biotechnology (Fujian Agriculture and Forestry University, Fuzhou, Fujian, China) and at the Sanming Academy of Agricultural Sciences (Sanming, Fujian, China). The plant material is commercially available at the two research institutions. The regenerated cuttings were propagated in sterile tissue culture on MS medium supplemented with benzyl adenine (2.0 mg/l), 2% sucrose and 0.6% agar at pH 5.8. The *Ec* bacteria were isolated from *Oncidium* seedling and conserved at the Sanming Academy of Agricultural Sciences (Sanming, Fujian, China), and the *Pi* fungal strain which was used for these studies is available from the Matthias-Schleiden-Institute, Plant Physiology (Friedrich Schiller University, Germany).

Co-cultivation of *Oncidium* with *Pi* in flasks were conducted as described previously [30]. Briefly, cuttings of about 6 cm height were transferred to fresh ½-strength Murashige-Skoog (MS) medium. After 10 day of acclimation, one agar block with *Pi* mycelium (or without, mock treatment) of 5 mm diameter was placed at a distance of 1 cm from the adventitious roots. The plants were cultured at a 16 h light/8 h dark photoperiod (100 μmol m^− 2^ s^− 1^) at 25 °C.

For pathogen resistance analysis, *Ec* was isolated and inoculated to *Oncidium* as described [3]. Briefly, the second leaves of *Oncidium* cuttings were punctured with a sterile tip, containing 2 μl of an *Ec* bacteria solution (OD_600_ = 1.0) in LB liquid medium, or LB medium alone (control). The plants were cultured on ½-strength MS medium at 25 °C in the flasks. The amounts of *Pi* and *Ec* DNA relative to the plant *ACTIN* DNA were detected by qPCR, primers were designed according to *Pi* EF-hand DNA (accession: FJ944820) and *Ec* 16S rDNA (assesion: KY020447) [3], respectively. Each PCR reaction was repeated three times with 3 independent biological samples. The primers for the target and reference genes are shown in Additional file 5: Table S3.

The levels of SA, JA, ETH and H_2_O_2_ in the leaves of plants co-cultivated with or without *Pi* were examined 24 h after *Ec* infection using the respective ELISA kits (ChunDu, China). Samples were extracted with phosphate buffer (100 mM, pH 7.2) in liquid nitrogen and measured with the Tecan M200 PRO plate reader (Switzerland) as described previously [103].

### Root dissection

Root samples were fixed as described previously [30]. Thin sections were cut by free hand and stained with lactophenol cotton blue solution [9] or acridine orange [104]. Sections were analyzed with an Olympus BX53 microscope system (Japan), fluorescence images were excited at 485 nm and detected at 540 nm before photography.

### Prediction of *R* genes from orchids and transcriptome analyses

The assembled transcriptome dataset from *Oncidium* Gower Ramsey was downloaded from the NCBI database (PRJNA428913). The *D. officinale* assembly and annotated genome V2.0 and the *P. equestris* assembly and annotated genome V5.0 were downloaded from the NCBI database (http://202.203.187.112/herbalplant; PRJNA192198) [33, 34]. *R* genes were predicted using HMMER v3 (http://pfam.xfam.org/) as described [105]. The NBS HMM file (PF00931) was downloaded from pfam (http://pfam.xfam.org/). R protein sequences from different orchid species were obtained using the raw NBS HMM with an E-value < 1^− 40^ after manual verification of the existence of NBS domains. Different orchid-specific NBS HMM files were generated, and these new orchid-specific HMM files were used to screen all proteins with E-value < 1^− 2^.

*R* genes were further analyzed based on manual verification of the existence of NBS domains. The annotation and identification of conserved domains were performed on BLAST2GO, sequence analyses were conducted with BLASTP against the NCBI nr database, and the conserved domains and the CC motif were analyzed using the InterProScan program. Additional motif analyses were conducted using MEME (meme-suite.org/tools/meme), the maximum number of motifs was set as 10, the minimum motif width as 6, the maximum motif width as 20, and the maximum sites per motif as 20.

### Alignment and phylogenetic analysis

The alignment and phylogenetic analysis were conducted as described [106]. Briefly, multiple alignment of the conserved NBS domain sequences (from P-loop to MDH-like motif) of the *Oncidium*, *Denddrobium* and *Phaleanopsis* R proteins were performed using ClustalW, a neighbor-joint phylogenetic tree was build using MEGA6.06, and 15 well-known R protein sequences from other species were also included. Bootstrap analysis was set with 1000 replicates to assess the stability of internal nodes. R protein sequences with < 10% intactness of the NBS domain were manually removed.

### Prediction of regulatory miRNAs for *R* genes in *Oncidium*

The *Oncidium R* gene sequences were submitted to psRNATarget (plantgrn.noble.org/psRNATarget/) and aligned to *Oncidium* miRNA sequences (accession: SRP031471) as described [30].

### qPCR expression analysis of *R* genes and their regulatory miRNAs in *Oncidium*

The leaves (*Ec*-infected or mock-treated leaves, or neighboring not infected leaves) of *Oncidium* plants pre-treated with *Pi* or mock-treated were collected for RNA isolation using isopropanol and LiCl methods as described [36]. Leaves treated with 1 mM SA and 0.1 mM methyl-JA (MeJA) for 24 h were also collected for RNA analyses. cDNA was synthesized using the PrimeScript™ RT reagent Kit with gDNA Eraser (RR047A; TAKARA) for *R* genes and miRcut (KR201; TIANGEN) for miRNAs, respectively. Expression analyses were performed using the ABI Q3 Real-Time PCR System with the SYBR Advantage qPCR Premix kit (639,676; Clontech). The qPCR reaction was performed in a total volume of 20 μl. Each reaction was repeated three times. The primers for the target and reference genes are shown in Additional file 5: Table S3.

## Supplementary information

**Additional file 1: Figure S1.** *Oncidium R* genes Blast2GO results; Multi-alignment of *Oncidium* R gene protein sequences. (PPTX 202 kb)

**Additional file 2: Figure S2.** *P. indica* colonization of *Oncidium* roots confers resistance against *E. chrysanthemi*. (PPTX 1961 kb)

**Additional file 3: Table S1.** *Oncidium R* genes Blast2GO results.

**Additional file 4: Table S2.** *R* gene sequences and their regulatory miRNAs.

**Additional file 5: Table S3.** The primer sequence information.

## Data Availability

All data generated or analyzed during this study will be freely available upon request to corresponding author**:** Wei Ye [E-mail:yewei922@qq.com] for scientific use.

## Abbreviations

*Ec*: *Erwinia chrysanthem*
ETH: Ethylene
ISR: Induced systemic resistance
JA: Jasmonic acid
MeJA: Methyl-jasmonate
*Pi*: *Piriformospora indica*
RT-PCR: Reverse transcription polymerase chain reaction
SA: Salicylic acid
