# Supplementary material for: Colonisation of Oncidium orchid roots by the endophyte Piriformospora indica restricts Erwinia chrysanthemi infection, stimulates accumulation of NBS-LRR resistance gene transcripts and represses their targeting micro-RNAs in leaves
Source: BMC Plant Biol. 2019 Dec 30;19:601. doi: 10.1186/s12870-019-2105-3 (PMC6937650; doi:10.1186/s12870-019-2105-3)
Supplement: Supplementary file 1 — Additional file 1: Figure S1. Oncidium R genes Blast2GO results; Multi-alignment of Oncidium R gene protein sequences. (PPTX 202 kb) [file 12870_2019_2105_MOESM1_ESM.pptx]

## Slide 1
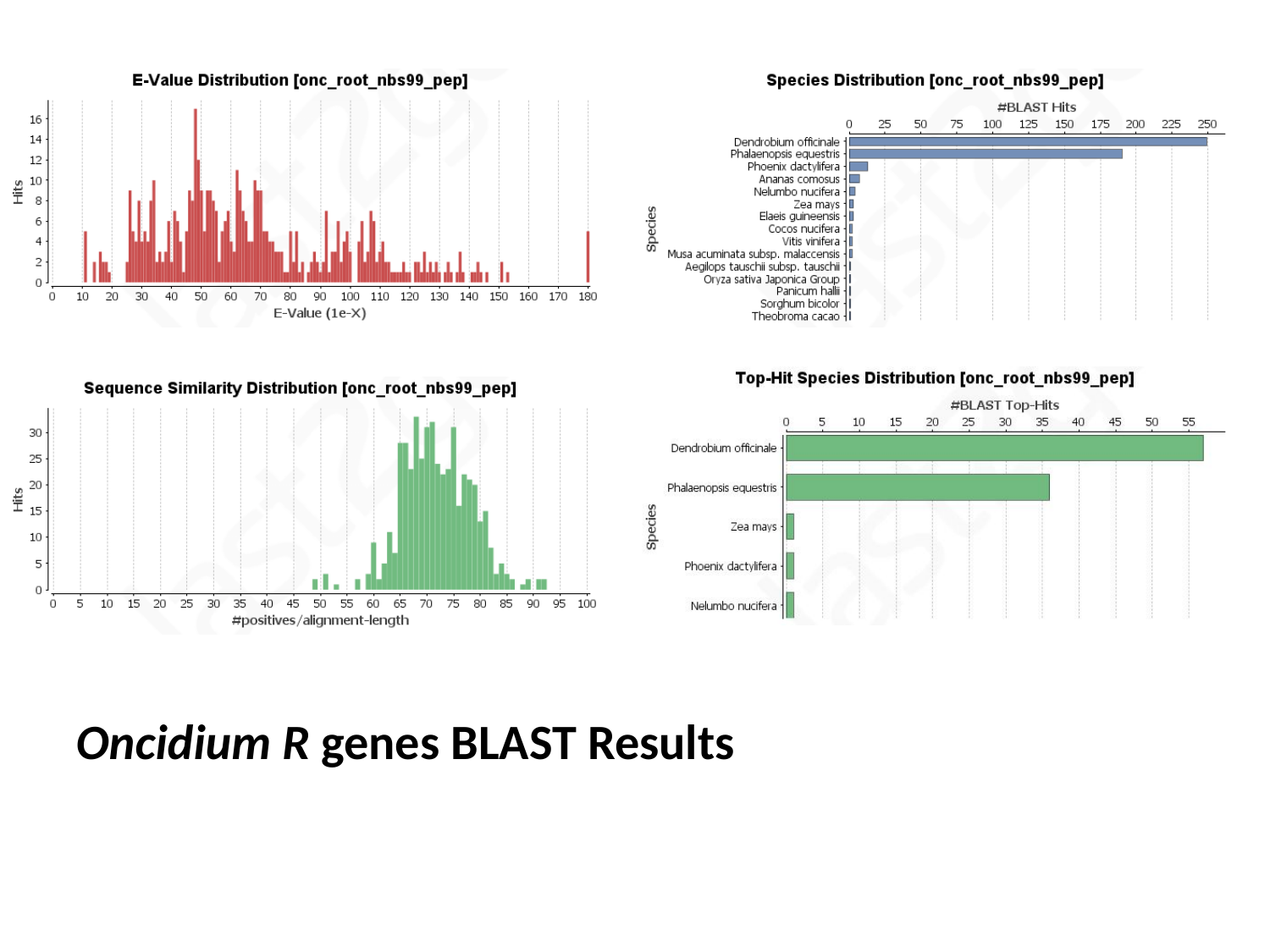

Oncidium R genes BLAST Results

## Slide 2
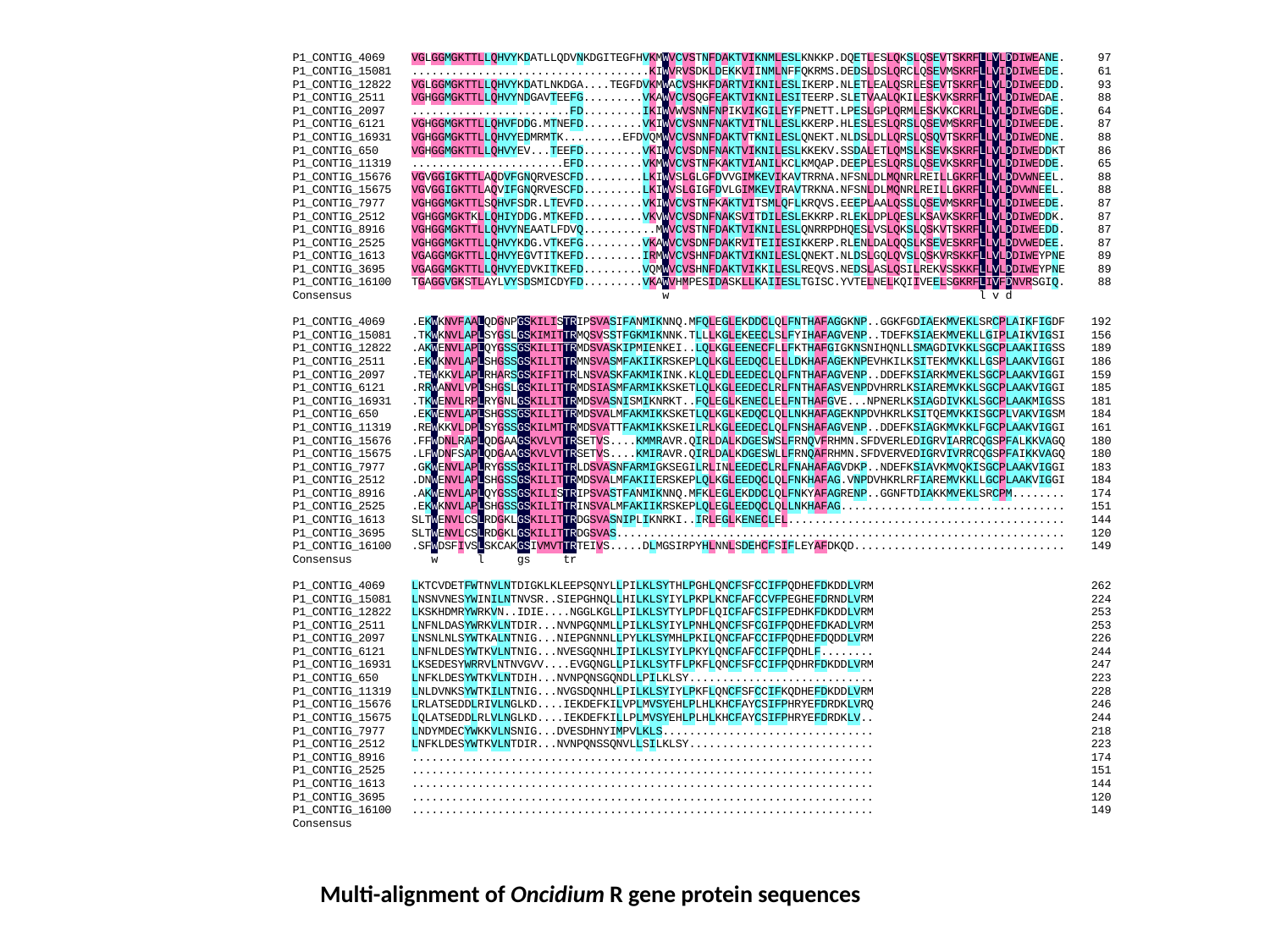

Multi-alignment of Oncidium R gene protein sequences
