# Supplementary material for: Colonisation of Oncidium orchid roots by the endophyte Piriformospora indica restricts Erwinia chrysanthemi infection, stimulates accumulation of NBS-LRR resistance gene transcripts and represses their targeting micro-RNAs in leaves
Source: BMC Plant Biol. 2019 Dec 30;19:601. doi: 10.1186/s12870-019-2105-3 (PMC6937650; doi:10.1186/s12870-019-2105-3)
Supplement: Supplementary file 2 — Additional file 2: Figure S2. P. indica colonization of Oncidium roots confers resistance against E. chrysanthemi. (PPTX 1961 kb) [file 12870_2019_2105_MOESM2_ESM.pptx]

## Slide 1
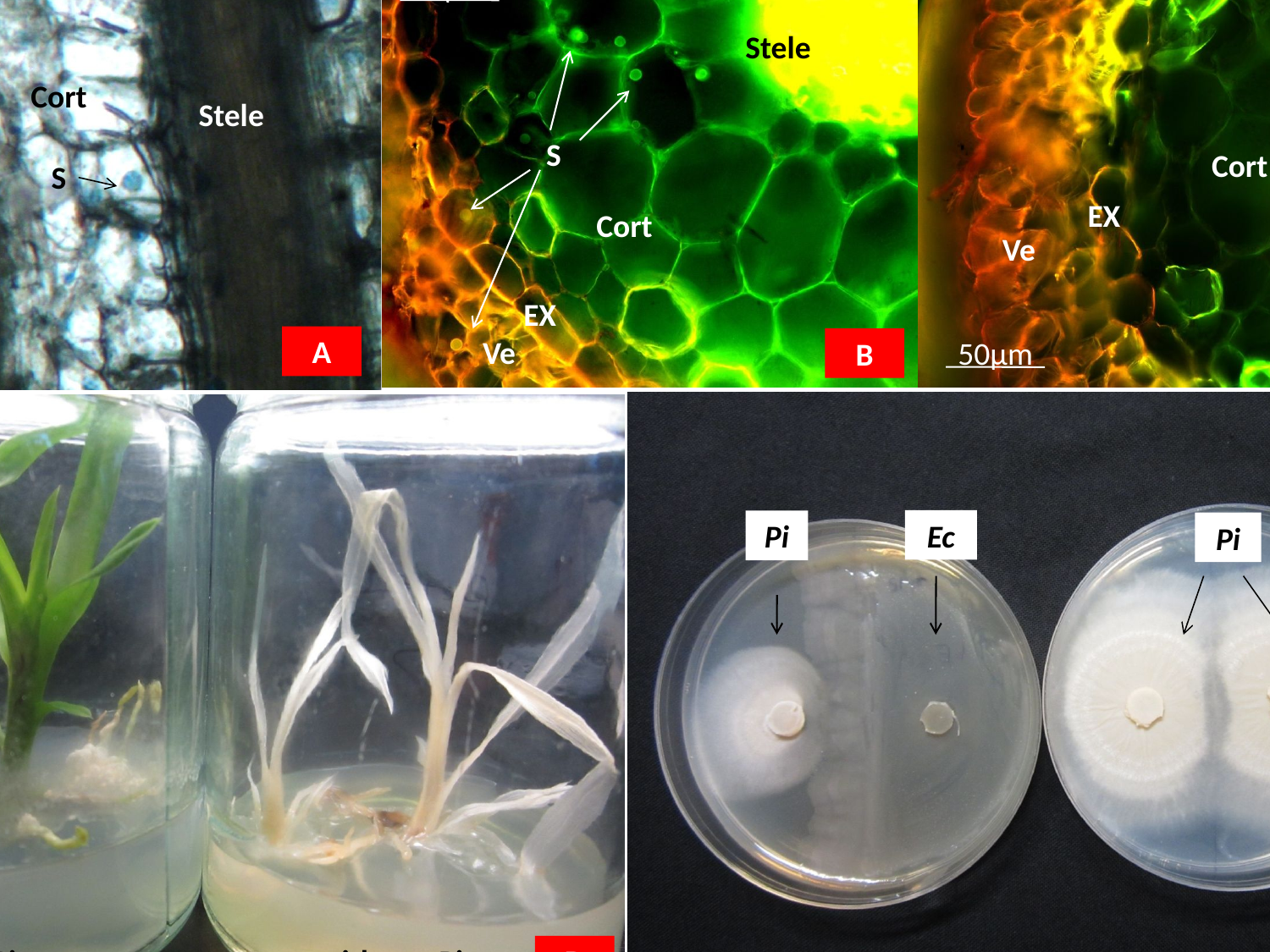

50μm
Stele
Cort
Stele
Stele
Ve
S
Cort
S
EX
Cort
Ve
EX
50μm
50μm
A
Ve
B
C
Ec
Pi
Pi
E
with Pi
without Pi
D

## Slide 2
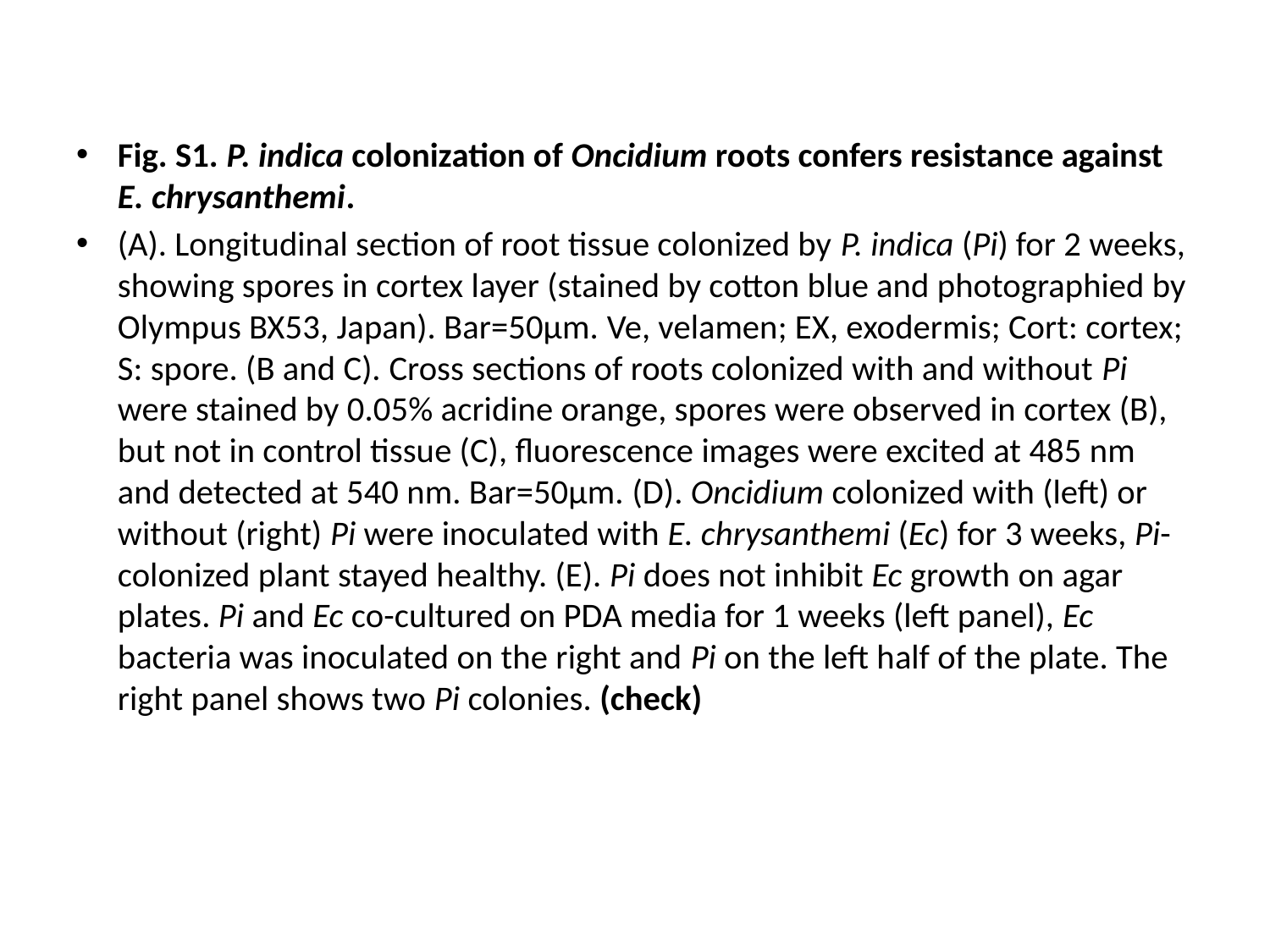

Fig. S1. P. indica colonization of Oncidium roots confers resistance against E. chrysanthemi.
(A). Longitudinal section of root tissue colonized by P. indica (Pi) for 2 weeks, showing spores in cortex layer (stained by cotton blue and photographied by Olympus BX53, Japan). Bar=50μm. Ve, velamen; EX, exodermis; Cort: cortex; S: spore. (B and C). Cross sections of roots colonized with and without Pi were stained by 0.05% acridine orange, spores were observed in cortex (B), but not in control tissue (C), fluorescence images were excited at 485 nm and detected at 540 nm. Bar=50μm. (D). Oncidium colonized with (left) or without (right) Pi were inoculated with E. chrysanthemi (Ec) for 3 weeks, Pi-colonized plant stayed healthy. (E). Pi does not inhibit Ec growth on agar plates. Pi and Ec co-cultured on PDA media for 1 weeks (left panel), Ec bacteria was inoculated on the right and Pi on the left half of the plate. The right panel shows two Pi colonies. (check)
